# Multiplex Assays for Analysis of Antibody Responses to South Asian *Plasmodium falciparum* and *Plasmodium vivax* Malaria Infections

**DOI:** 10.3390/vaccines12010001

**Published:** 2023-12-19

**Authors:** Elizabeth O. Momoh, Sonam K. Ghag, John White, Devaraja G. Mudeppa, Pradipsinh K. Rathod

**Affiliations:** Department of Chemistry, University of Washington, Seattle, WA 98195, USA; emomoh@uw.edu (E.O.M.); sonamg@uw.edu (S.K.G.); jwhite3@uw.edu (J.W.)

**Keywords:** malaria antigens, cell-free expression, functionalized beads, antigen adsorption, clinical samples, multiplex assays

## Abstract

Malaria remains a major global health challenge, causing over 0.6 million yearly deaths. To understand naturally acquired immunity in adult human populations in malaria-prevalent regions, improved serological tools are needed, particularly where multiple malaria parasite species co-exist. Slide-based and bead-based multiplex approaches can help characterize antibodies in malaria patients from endemic regions, but these require pure, well-defined antigens. To efficiently bypass purification steps, codon-optimized malaria antigen genes with N-terminal FLAG-tag and C-terminal Ctag sequences were expressed in a wheat germ cell-free system and adsorbed on functionalized BioPlex beads. In a pilot study, 15 *P. falciparum* antigens, 8 *P. vivax* antigens, and a negative control (GFP) were adsorbed individually on functionalized bead types through their Ctag. To validate the multiplexing powers of this platform, 10 *P. falciparum*-infected patient sera from a US NIH MESA-ICEMR study site in Goa, India, were tested against all 23 parasite antigens. Serial dilution of patient sera revealed variations in potency and breadth of antibodies to various parasite antigens. Individual patients revealed informative variations in immunity to *P. falciparum* versus *P. vivax*. This multiplex approach to malaria serology captures varying immunity to different human malaria parasite species and different parasite antigens. This approach can be scaled to track the dynamics of antibody production during one or more human malaria infections.

## 1. Introduction

*Plasmodium falciparum (Pf)* and *P. vivax (Pv)* are primary causative agents of human malaria. Together, they are responsible for about 250 million malaria cases yearly, resulting in over 600,000 deaths [1]. Despite the deployment of efficacious antimalarials, insecticides, and bed nets, the decline in the worldwide parasite burden has stalled in recent years [1,2,3]. Repeated exposure to malaria parasites produces antibody responses to many antigens and confers naturally acquired immunity [4,5,6]. Understanding variations in serum antibody responses in malaria patients can offer insights into serological markers for submicroscopic infections, activated hypnozoites, and protective immunity. Multiplexed slide-based protein arrays offer an important approach for malaria parasite antigen prioritization [6,7,8,9,10,11,12]. A single glass-based array can report on differential antibody binding from patient sera to hundreds of parasite antigens in parallel [8,13,14].

As part of a global collaboration through the US NIH International Centers of Excellence for Malaria Research (ICEMRs), malaria protein arrays have been used to study immunity against *P. falciparum* and *P. vivax* [6,11,12,14,15,16,17,18,19,20,21]. Traditionally, protein arrays have been fabricated using unpurified malaria antigens spotted directly onto glass slides [10,11]. This is often in the absence of antigen validation for correct folding or purity. Thus, important disease biomarkers may be unreactive [6,10,11,22,23,24,25]. On the other hand, slide-based arrays do offer the capacity for versatile fabrication and customization for specialized applications.

More recent bead-based methods for dissecting broad immune responses include the AlphaScreen [26] and BioPlex systems [27,28]. Using spectrally unique beads carrying unique antigens, the BioPlex platform is well-suited for multiplexed interrogating many antigens simultaneously [29]. Previous multiplexed bead-based methods have detected malaria patient antibody responses [30,31,32,33,34,35,36,37,38]. However, in these earlier studies, malaria antigen panels had limited representation. In addition, standard antigen coupling to beads was preceded by laborious purification of individual antigens from translation lysates, which can limit the breadth of larger antigen panels.

In some regions, such as South and Southeast Asia, there is a need for antigen arrays that include *Pf* and *Pv* antigens, particularly polymorphisms seen in local infected communities. In the present study, a streamlined bead-based protein array platform is described for the dissection of naturally acquired immunity in malaria patients. In a pilot study, recombinant parasite antigens were selectively adsorbed from translated lysates onto beads modified with an affinity reagent. To demonstrate the versatility of this approach, such modified beads successfully measured different antibody titers against 23 *Pf* and *Pv* antigens from 10 patient sera. Expansion of these methods will help dissect patient antibody profiles, identify multiple important malarial serological markers for infection and disease, and help improve our understanding of differential immunity in malaria patients.

## 2. Materials and Methods

### 2.1. Ethical Statement

The human subject protocol and consent forms for enrolling Plasmodium-infected patients in this study at Goa Medical College and Hospital (GMC) were reviewed and approved by the Institutional Review Boards of the Division of Microbiology and Infectious Diseases (DMID) at the U.S. National Institute of Allergy and Infectious Diseases (approval DMID 11-0074), the University of Washington (approval 42271/1192), as well as the Institutional Ethics Committee (IEC) at Goa Medical College Hospital, Bambolim, Goa, India.

### 2.2. Sample Collection

Patient samples were collected as part of a US National Institutes of Health (NIH)-sponsored activity on the Malaria Evolution in the South Asia-International Center of Excellence for Malaria Research (MESA-ICEMR). Plasma samples were collected from symptomatic malaria-positive patients at Goa Medical College (Goa, India). Written informed consent was obtained from all volunteers. A detailed description of the study site, enrollment, and sample processing has been published elsewhere [39]. Naïve human sera from malaria-free individuals (BioChemed, Winchester, VA, USA) were used as a negative control.

### 2.3. Antigen Constructs

Malaria antigen genes were designed to carry a 5′ XhoI restriction site followed by a start codon (ATG) and a Flag-tag (DYKDDDDK) coding sequence. On the 3′ of each antigen, a Ctag (GAAEPEA) coding sequence and a stop codon (TGA) were followed by an EagI restriction site. Codons of the antigen constructs were optimized on the GeneArt server (GeneArt, Thermo Fisher Scientific, Waltham, MA, USA) to support expression in a wheat-based protein expression system [39]. Optimized genes were chemically synthesized (GeneArt) and subcloned into a cell-free vector as earlier described [40]. Plasmids were purified using Qiagen kits (Qiagen, Germantown, MD, USA), and the resulting DNA products were validated by Sanger sequencing.

### 2.4. In Vitro Transcription

In vitro transcription of malaria antigens was carried out as previously described [40,41]. For small-scale batch-method protein expression, 50 µL reactions contained 4 µg of plasmid DNA, transcription buffer (80 mM HEPES-KOH pH 7.8, 16 mM magnesium acetate, 2 mM spermidine, 25 mM β-mercaptoethanol), 10 units of ribonuclease inhibitor (Promega, Madison, WI, USA), 50 units of SP6 RNA polymerase (New England Biolabs (NEB), Ipswich, MA, USA), and 3 mM each of GTP, ATP, CTP, and UTP. The transcription mixture was incubated at 37 °C for 4 h, followed by purification using Microspin G25 columns (GE Healthcare, Chicago, IL, USA). For scaled-up method-based translations, a 600 µL transcription mixture was used that contained 45 µg of plasmid DNA, 1.5× transcription buffer, 100 units of ribonuclease inhibitor (Promega), 900 units of SP6 RNA polymerase (NEB), and 4.5 mM each of GTP, ATP, CTP, and UTP. The mixture was incubated at 37 °C for 4 h, followed by centrifugation at 25 °C at 10,000× *g* for 5 min. The mRNA in the supernatant was used for wheat germ cell-free translations.

### 2.5. Cell-Free Expression of Malarial Antigens

For the initial verification of expressions, a batch translation method was used for generating radiolabeled antigens. The cell-free translation of purified mRNA was carried out using an in-house preparation of wheat germ lysate. A 50 µL translation contained 10 µL mRNA, 4 units of wheat germ lysate, one µg creatine kinase, 40 units of ribonuclease inhibitor, and a custom protein expression buffer (30 mM HEPES-KOH pH 7.8, 100 mM potassium acetate, 2.7 mM magnesium acetate, five mM DTT, 0.4 mM spermidine, 1.2 mM ATP, 0.25 mM GTP, 16 mM creatine phosphate, 0.3 mM concentration of 19 amino acids ((-)leucine), and 29.6 kBq ^14^C leucine (Moravek, Brea, CA, USA)). Translation reactions were incubated at 26 °C for 4 h, followed by centrifugation at 10,000× *g* at 4 °C. Soluble fractions were boiled with Laemmli Sample buffer with 2% β-mercaptoethanol (β-me) and resolved by sodium dodecyl sulfate-polyacrylamide gel electrophoresis (SDS-PAGE). The gels were dried for two hours and then exposed to a phosphor imager screen (GE Healthcare). The screen was scanned on a GE Typhoon FLA 9000 Gel Imager to visualize the position of radiolabeled proteins. For scaled-up translations, 500 µL of mRNA was mixed with an equal volume of wheat germ extract (to give a final OD of 120 A_260_ units), 80 µg creatine kinase, 40 units of ribonuclease inhibitor (Promega), and 0.1% TritonX-100. To each well of a six-well flat bottom culture plate, 10 mL of protein expression buffer was added. A translation reaction mixture (one mL) was injected into the bottom of each well, creating two separate layers. Plates were incubated at 26 °C for 20 h.

### 2.6. Functionalization of Luminex Beads with Anti-Ctag Antibodies

Carboxylated color-coded magnetic Luminex beads were further functionalized by covalent coupling of the anti-Ctag antibody by following the manufacturer’s instructions. In a typical coupling reaction, one million beads (Luminex, Austin, TX, USA) were vortexed and sonicated, then kept on the magnetic stand to pipette out the buffer solutions. The beads were resuspended in 50 µL of 0.1 M MES pH 4.5 activation buffer containing 5.5 mg per ml each of N-hydroxysulfosuccinimide (sulfo-NHS, Thermo Fisher Scientific) and carbodiimide (EDC, Thermo Fisher Scientific). After 25 min of activation in the dark at room temperature, beads were washed on a magnetic stand twice with 0.1 M MES pH 5.0. Next, in a series of 100 µL reactions, one million activated beads were mixed with 0.1 M MES pH 5.0 buffer containing 0 to 75 µg biotinylated anti-Ctag antibody (Thermo Fisher Scientific) and incubated in the dark at room temperature for two hours with end-to-end rotation. Anti-Ctag antibody functionalized beads (AFBs) were washed three times with storage buffer PBS-TBN (PBS, 0.1% BSA, 0.02% Tween20, and 0.05% sodium azide) on a magnetic stand and stored in the same buffer at 4 °C. The AFBs were counted using a hemocytometer.

Covalent attachment of biotinylated anti-Ctag antibody to beads was confirmed using streptavidin-conjugated R-phycoerythrin (SAPE, Thermo Fisher Scientific) on a BioPlex 200 system (Bio-Rad Laboratories, Harcules, CA, USA). In a 96-well plate, triplicates of 1000 counts of beads (from each concentration of AFBs per well) were added. All binding assays in this report were performed in 100 µL of buffer solutions unless otherwise mentioned. The AFBs were separated from the storage buffer by placing the plate on a handheld magnetic washer (Luminex) for 90 s. Microspheres were washed once with assay buffer (0.1% BSA in PBS), followed by the addition of 100 µL of serially diluted SAPE. The plate was incubated in the dark at room temperature for 30 min with orbital shaking at 800 RPM. The unbound SAPE was washed from the AFBs once with 100 µL assay buffer. The AFBs were resuspended in 100 µL assay buffer and read on a BioPlex 200.

### 2.7. Adsorption of Ctagged PfMSP1-42 on Functionalized Beads from Wheat-Translated Lysates

Optimization of malarial antigen adsorption to AFBs was carried out using *Pf*MSP1-translated lysates. The *Pf*MSP1-42 wheat translated lysate (WGL) from the bilayer reaction was centrifuged at 20,000× *g* for 10 min at 4 °C, followed by a collection of *Pf*MSP1-42 in supernatants of translated lysates for further studies. To a series of 15 mL tubes containing one million AFBs, 0 to 9 mg (0 to 5 mL of WGL) of supernatant of *Pf*MSP1-42 WGL was added, and the final volume was adjusted to 7.5 mL with binding buffer (20 mM Tris pH 7.5, 100 mM NaCl, 0.05% TritonX-100). The mixtures were incubated in the dark at room temperature, with end-to-end rotation for two hours. The tubes were spun at 4000× *g* for 5 min to separate beads from unbound proteins. The beads were washed thrice with binding buffer and transferred to 1.5 mL microcentrifuge tubes. The beads were washed again with storage buffer (Phosphate Buffered Saline pH 7.4, with 0.045% Tween, 0.1% BSA, and 0.05% Azide). The *Pf*MSP1-42 adsorbed AFBs were placed in a storage buffer at 4 °C, and the AFB count was determined using a hemocytometer.

### 2.8. Validation of Adsorbed PfMSP1-42 on Anti-Ctag Antibody Functionalized Beads

Adsorbed *Pf*MSP1-42 protein on AFBs was validated in a multiplex assay using anti-flag antibodies on a BioPlex 200 system. In a 96-well plate, triplicates of 2000 counts of each set of *Pf*MSP1-42 adsorbed AFBs were mixed with 100 ng per ml of anti-flag rabbit antibodies (Abcam, Waltham, MA, USA) in assay buffer in triplicates. The plate was incubated in the dark at room temperature for one hour with orbital shaking at 800 RPM, followed by three washes with assay buffer. Antigen-coated AFBs were resuspended in 100 µL assay buffer containing 100 ng per ml of PE-conjugated goat F(ab’)2 anti-rabbit antibodies (Thermo Fisher Scientific). The plate was incubated for 30 min with orbital shaking, followed by three five-minute washes with assay buffer. Fluorescence was quantified on a BioPlex 200 system as described earlier.

Large-scale adsorption of individually translated malaria antigens from WGL on separate bead types was carried out in a 15 mL reaction volume. To overcome the variations in antigen expression levels in the wheat germ system and differential coating on beads, twice the volume of optimized antigen-translated lysates was used to adsorb onto functionalized beads. One million AFBs were typically mixed with 10 mL of WGL and 5 ml of binding buffer. After washing off the wheat germ proteins three times in the binding buffer, the malaria antigen-adsorbed AFBs were counted on a hemocytometer. They were pooled to obtain an approximately equal number of antigen-adsorbed AFBs per ml of storage buffer. Antigen-coated bead types were visualized using anti-flag rabbit antibody and PE-conjugated goat anti-rabbit antibody as described earlier.

### 2.9. Patient Antibody Binding Studies in Multiplex Assay

Patient sera were initially diluted 50-fold in the assay buffer. These 50-fold diluted sera were subjected to 5 sequential 3-fold dilutions. Each malaria antigen-adsorbed bead set, and one GFP-adsorbed bead set, was aliquoted to obtain 1000 beads per type per well. Beads were washed twice with assay buffer (0.1% BSA in PBS). Each serially diluted serum (100 µL) was added to three wells and incubated for an hour at room temperature. After washing off the unbound sera, 100 µL of goat anti-human IgG-PE (Millipore Sigma, Burlington, MA, USA), at 6 µg/mL, was added to each well and incubated for 30 min as earlier. The microspheres were washed three times in assay buffer, resuspended in 100 µL of assay buffer, and read on a BioPlex 200 system. To calculate half maximal titers, saturating binding curves of patient antibodies to target antigens were generated on Prism 10 (GraphPad, San Diago, CA, USA). By utilizing the equation for one site binding models (Y = Bmax × X/(Kd + X)), half-maximal binding values (Kd) were calculated. Reciprocals of these Kd values are called reciprocals of half-maximal titers (MT50). Half-maximal values were not calculated and considered negative for the antigens that displayed less than 200 mean fluorescence intensity (MFI), and no change in fluorescence was observed against all tested sera dilutions.

## 3. Results

### 3.1. Antigen Selection

**Figure 1:**
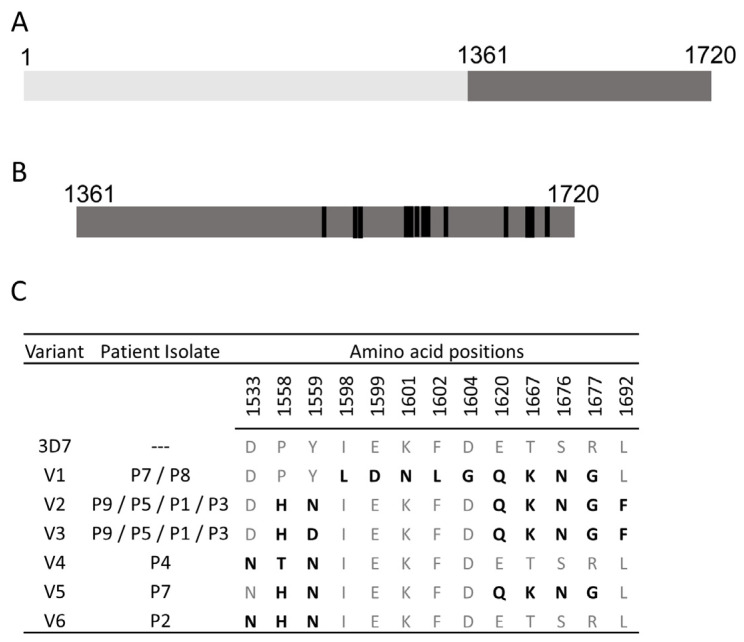
*Pf*MSP1-42 (PF3D7_0930300) polymorphisms in Goan clinical isolates. (**A**) Schematic representation of full-length *Pf*MSP1 and (**B**) its C-terminal portion (*Pf*MSP1-42). The numbers on the schematics indicate the amino acid positions. The black bars on *Pf*MSP1-42 indicate the position of mutations. (**C**) Amino acid changes in 3D7 *Pf*MSP1-42 variants by position. The highlighted amino acids differ from the 3D7 sequences.

In order to identify malaria serological markers that define submicroscopic infections, activation of hypnozoites, and protective immunity in the Indian subcontinent, the following reports were considered. In a MESA-ICEMR initiated *P. falciparum* whole genome sequencing project, it was found that clinical isolates from Indian patients were genetically distinct compared to the isolates from the rest of the world [42]. The Indian malaria parasites displayed distinct polymorphisms in known drug targets, serological markers, and vaccine candidates. To assess the impact of antigenic polymorphisms on patient antibody binding, in the present report, clinical isolates from Goa were considered. Based on mutations found on *Pf*MSP1 sequences from nine Goan clinical isolates, six different variants of *Pf*MSP1, each with a distinct set of mutations, were constructed (Figure 1). As all identified mutations from these clinical isolates were concentrated at the amino acid position from 1361 to 1692 of *Pf*MSP1 (Figure 1A), the 42 kDa portion at the C-terminus of antigen *Pf*MSP1-42s (Figure 1B) was constructed. Some variants were found in more than one patient, whereas others were unique to a patient (Figure 1B). In other recent serological studies from India [12,16], top reactive *P. falciparum* and *P. vivax* serological markers that were distinct to India compared to the rest of the world [26,43,44,45] were highlighted From this list, five antigens, each from *P. falciparum* and *P. vivax*, were considered for further studies. Overall, in this pilot study, 15 *P. falciparum* antigens, 8 *P. vivax* antigens, and negative control (GFP) were chosen (Table 1). Of the 15 *P. falciparum* antigens, 8 were based on truncated constructs of *Pf*MSP1 (*Pf*MSP1-19 and *Pf*MSP1-42) and its variants; 5 were based on the recently reported top reactive *P. falciparum* serological markers from India, and the remaining 2 are *Pf*AMA1 and *Pf*Rh5. Of the eight *P. vivax* antigens, two were truncated constructs of *Pv*MSP1s (*Pv*MSP1-19 and *Pv*MSP1-42); and five were top reactive *P. vivax* markers from India and a *Pv*AMA1.

### 3.2. Rapid Generation of Malaria Antigen-Coated BioPlex Beads

To overcome the limitations of the purification of malaria antigens in serological studies, a method was designed for the selective adsorption of malaria antigens onto beads from translated lysates. A commercially available CaptureSelect™ biotinylated anti-Ctag antibody (anti-Ctag ab), which is a Camelid single-domain 13 kDa antibody fragment (VHH), was utilized to achieve this possibility. Initially, the versatility of anti-Ctag ab was tested by purifying Ctagged *Pf*MSP1-42 and GFP on resin coupled with this single-domain antibody. After passing through the *Pf*MSP1-42 or GFP translated lysates onto the anti-Ctag ab column, a simple wash with buffer solutions and gentle elution with a competing peptide yielded more than 80% pure *Pf*MSP1-42 antigen (supplemental data; Figure S1B). Such efficient capture of the malaria antigens from wheat-translated lysates with minimum background, higher purification yields, and purity was difficult to achieve using conventional purification resins. The quality of the wheat germ-expressed Ctagged *Pf*MSP1-42 was further verified by generating antibodies in rabbits. On a Western blot, the rabbit-generated anti-*Pf*MSP1-42 antibody detected a single band of 190 kDa protein from 3D7 *P. falciparum* lysates (supplemental data; Figure S1C), corresponding to the size of full-length *Pf*MSP1. The anti-*Pf*MSP1 rabbit antibody also displayed parasite growth inhibitory activity in cell cultures (supplemental data; Figure S1D). Next, a preliminary study assessed the ability of the anti-Ctag ab coupled to BioPlex beads in efficiently adsorbing Ctagged malaria antigen (supplemental data; Figure S2). When a purified *Pf*MSP1-42 is attached to a bead, either chemically (Figure S2A), adsorbed on an anti-Ctag ab functionalized bead (Figure S2C), or *Pf*MSP1-42 adsorbed directly from the translated lysates to an anti-Ctag ab functionalized bead (Figure S2D), a similar MFI was observed on the BioPlex 200. These results suggest the potential ability of the BioPlex bead functionalized with anti-Ctag ab in adsorbing Ctagged proteins from translated lysates with minimal interference from background proteins and chemicals. These results prompted us to optimize antigen adsorption and to expand the study of multiple malaria antigens from translated lysates.

**Figure 2:**
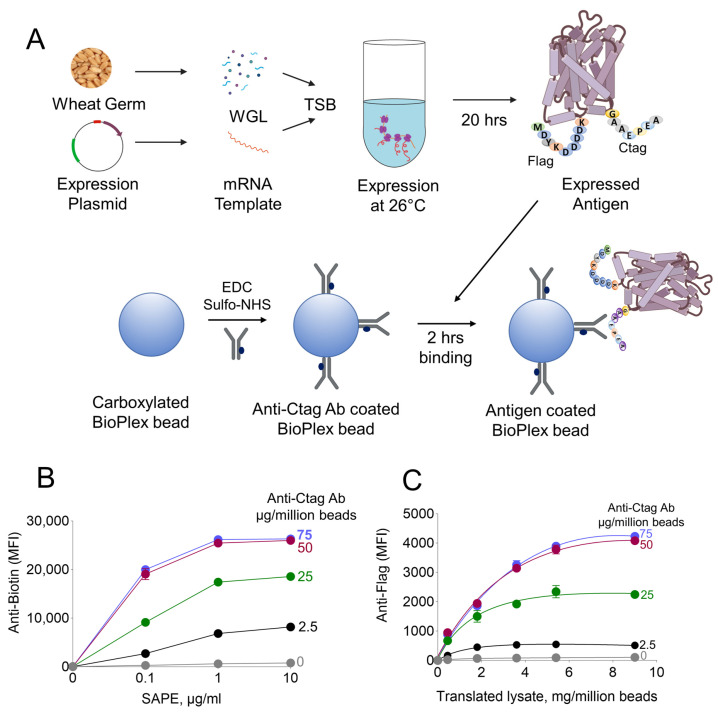
Overview of a methodology for a rapid generation of antigen-coated beads. (**A**) Illustration depicting the process of malaria antigen expression in wheat germ cell-free expression system and the selective adsorption onto functionalized beads. (**B**) Saturation curves showing the mean fluorescence intensities (MFI) of biotinylated anti-Ctag antibody chemically attached to BioPlex beads employing streptavidin-phycoerythrin antibody conjugate (SAPE) for detection and quantification. (**C**) Titration for optimal adsorption of a *Pf*MSP1 (PF3D7_0930300) onto functionalized BioPlex beads utilizing anti-Flag antibodies.

A generalized scheme was designed for the wheat germ cell-free expression of malaria antigens and their selective adsorption on anti-Ctag antibody-coated beads (Figure 2A). Malaria antigens were designed to have an N-terminal flag-tag and a C-terminal Ctag. This allowed us to conveniently track binding antigens to beads. Carboxylated BioPlex beads were further functionalized with various quantities of anti-Ctag antibodies using EDC-NHS chemistry. The chemically attached ant-Ctag antibody was quantified using streptavidin-conjugated R-PE on the BioPlex200 system. A 50 µg of anti-Ctag ab at 0.5 mg/mL was sufficient for saturating the chemical attachment to one million beads (Figure 2B). Next, anti-Ctag functionalized beads were tested for their capacity to adsorb *Pf*MSP1 antigen. The *Pf*MSP1 adsorbed to anti-Ctag coated beads was monitored by anti-flag antibodies. Maximum adsorption of *Pf*MSP1 was achieved with nine mg equivalent of wheat germ lysate per one million anti-Ctag coated beads (Figure 2C).

### 3.3. Parallel Adsorption of Malaria Antigens on Functionalized Beads and Their Validation

**Figure 3:**
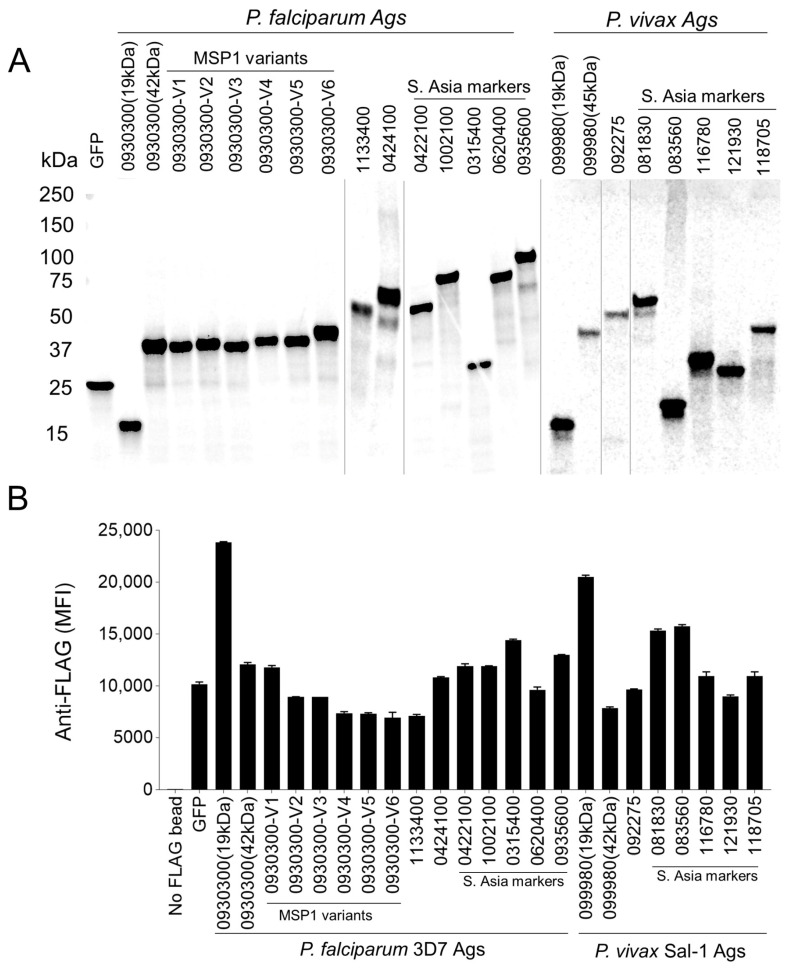
Validation of malaria antigen expression and adsorption onto functionalized beads. (**A**) An autoradiogram verifies the quality of malaria antigens expressed in the wheat cell-free system. (**B**) Confirmation of malaria antigen adsorption onto beads using anti-flag antibodies.

The quality of the wheat-expressed 23 antigens and a negative control, GFP, was confirmed on an autoradiogram (Figure 3A). All antigens from soluble fractions of translated lysates appeared as single bands with expected masses, which was consistent with a previously adapted codon optimization strategy for minimizing protein fragmentation [39]. Next, a scaled-up bilayer reaction method of cell-free expression of 23 antigens was carried out individually in six-well plates. Supernatants of translated lysates were allowed to adsorb to functionalized bead sets separately. Each Ctagged antigen-adsorbed bead type was pooled and their occupancy was tracked using anti-flag antibodies (Figure 3B). A mean fluorescence intensity of 5000 or more, well above the noise level, corresponded to the maximum adsorption of each antigen to their respective beads.

### 3.4. Patient Antibody Levels to P. falciparum and P. vivax Antigens

**Figure 4:**
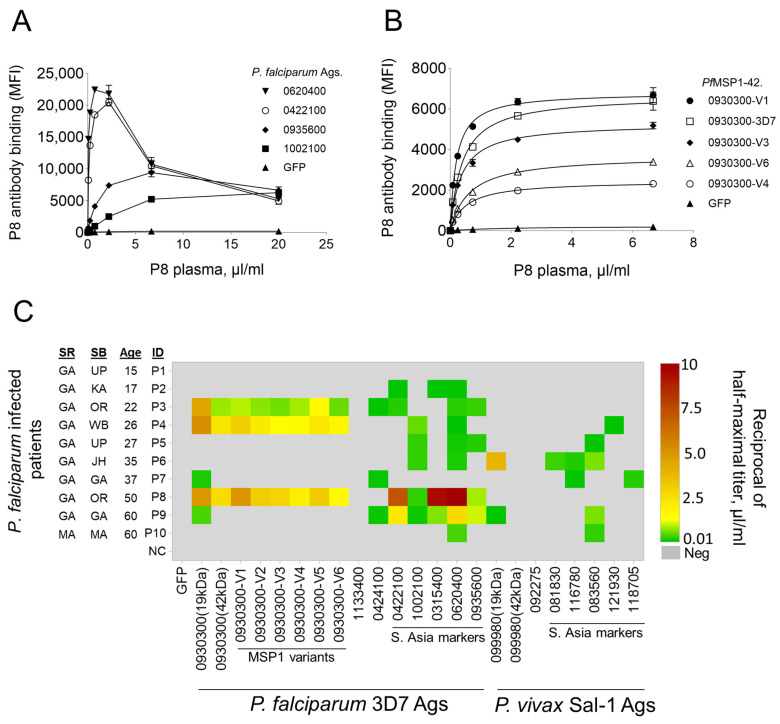
The magnitude of patient IgG responses to malaria antigens. (**A**) Assessment of IgG antibody levels against *P. falciparum* and *P. vivax* antigens by testing serially diluted patient serum. (**B**) Representative binding curves generated for the calculation of reciprocals of Half-Maximal titers. (**C**) Seroreactivity profiles for *P. falciparum* and *P. vivax* antigens. Patient samples are organized in ascending order of age, with migration status determined by their State of Birth (SB) and State of Residence (SR). GA-Goa; UP-Utter Pradesh; KA-Karnataka; OR-Orissa; WB-West Bengal; JH-Jharkhand; MA-Maharashtra. Additional information regarding these clinical patient samples is reported in Supplemental Table S1.

In our preliminary serological studies, a single-point sera dilution did not always reveal the breadth of patient antibodies for all antigens. In this report, seven-point serial diluted sera were used to uncover the true variation of antibody reactivity against all antigens in each patient sample. In a multiplex assay, a pool of 23 antigens was tested in triplicates against seven sera dilutions from each patient sample. This was necessary because each antigen-antibody pair had a unique combination of affinity versus protein abundance and thus varied between patients. The IgG from patient 8 (P8), for example, displayed different levels of sera dilutions for optimal binding to various antigens (Figure 4A). The P8 IgG bound to *P. falciparum* antigens 0620400 and 0422100 optimally at 0.74 and 2.2 µL/mL, respectively. Conversely, the same patient antibodies bound optimally at 20 and 6.6 µL/mL to *P. falciparum* antigens 0935600 and 1002100. Interestingly, at higher serum dilutions, low-level antibodies to some *P. falciparum* antigens (0935600 and 1002100) hovered around background levels, and lower serum dilutions inhibited the binding of patient antibodies to other antigens (0620400 and 0930300 (19kDa)) optimally. Similarly, testing serially diluted sera samples against variants of *Pf*MSP1-42 helped to uncover patient-specific antibody binding preferences (Figure 4B). The reciprocals of half-maximal titers (MT50) were calculated from non-linear binding curves of patient IgG to antigens. The antibodies from P8 preferred the *Pf*MSP1-V1 variant with MT50 of 5 µL/mL compared to any other variant (MT50: 1.6 to 3.1 µL/mL), including the 3D7 construct (MT50: 2.56 µL/mL). MESA-ICEMR enrolled patient samples (Supplemental Table S1) displayed MT50s in the 0.01 to 10 µL/mL range for all antigen constructs (Figure 4C). Younger patient samples, P1 (age 15) and P2 (age 17) showed zero to low levels of antibodies only to India markers (0422100, 01315400, and 0620400) and not to *Pf*MSP1, *Pf*Rh5, or *Pf*AMA1. Of the remaining eight *P. falciparum-*infected samples (age 20 and above), IgG from only five patients bound to common *P. falciparum* antigens like *Pf*MSP1 (0930300) *Pf*Rh5 (0424100). However, IgG from these patients bound to at least one of five tested Indian markers. Incidentally, patients who migrated from Orissa (OR) had IgG for six (P3) and seven (P8) of the nine tested *P. falciparum* antigens. Similarly, a patient (P4) who migrated from West Bengal (WB) had IgG for four of the nine tested antigens. Although all 10 tested patient samples were confirmed (Microscopy, RDT, and PCR) positive for *P. falciparum* infections (Supplemental Table S1), 6 patients had IgG to *P. vivax* India markers, indicating prior exposure to *P. vivax*.

## 4. Discussion

Malaria parasites invade human red blood cells using a combination of multiple ligands. There appears to be a plethora of antibody responses to these surface antigens as well as to the intracellular proteins. Identifying serological markers that define submicroscopic infections, activation of hypnozoites, and defining protective immunity could greatly help malaria control efforts. There have been collaborative efforts to identify dominant *P. vivax* serological markers [43,44]. These serological studies are from malaria transmission regions but exclude the Indian subcontinent. In contrast, early serological studies from India indicated a different set of highly seroreactive *P. vivax* antigens [12,16]. Similar differences in the *P. falciparum* seroreactive antigens were also reported from different geographical regions [12,16,26,45]. Additionally, the impact of antigenic polymorphisms on evading the immune response remains to be understood.

The present study demonstrates a simple but versatile bead-based antigen assay that can be deployed to quantify antibody levels against malaria antigens in regions of India with both *Pf* and *Pv* transmission. Here, we combine the sensitivity and specificity of bead-based technology with a powerful wheat cell-free antigen expression system and a freshly developed protocol to functionalize beads to adsorb antigens from translated lysates. The presented multiplex approach offers flexibility to adjust the serological markers quickly to meet the local transmission settings. Antigen-adsorbed beads are stable for over 90 days when stored at 4 °C. Additionally, during these stability studies, the drift of antigens between the beads was not observed (supplemental data: Figure S3). This stability and lack of exchange of antigens between beads is critical when pooled beads are stored and shipped. Antigen-translated lysates from each ml of wheat germ extract are enough to cover over three million beads. Considering 1000 beads per assay, about 3000 assays can be performed. Bypassing the antigen purification step makes the method easy to integrate with hundreds of antigens in parallel.

Employing serially diluted sera in preference to commonly implemented single-point dilution serological tests, we uncovered the absolute levels of patient IgG to all studied antigens. For example, *Pf*MSP10 (0620400) had the highest level of antibodies (MT50: 10 µL/mL) in P8 and the lowest level of antibodies (MT50: <0.02 µL/mL) in P4. Similarly, *Pf*MSP1 (0930300) displayed a 2- to 10-fold difference in binding to patient antibodies compared to its variants. Since the *Pf*MSP1-42-V1 construct was designed from sequences of P8 parasite isolate and had unique mutations compared to other constructs (Figure 2B), it is possible that the patient was infected and reinfected with parasite carrying V1 sequences, which agrees with a previous report on allele-specific *Pf*MSP1 antibody responses in mice studies [46]. This reasoning is supported by the least preference from P8 antibodies to construct V4 and V6 with no overlapping V1 mutations. The *Pf*MSP1-42 variant preference differences were not observed in patients P3 and P4. Six of the ten *P. falciparum*-infected samples showed low antibodies to *P. vivax* antigens, especially those India dominant markers, indicating prior exposure to *P. vivax*.

## 5. Conclusions

The multiplexed approach for serological studies presented here can help uncover the variations of antibody levels to many antigens from different human malaria parasites. Serological data on geographically dominant antigens and their variants combined with clinical data can help to detect submicroscopic infections and define protective immunity.

## Figures and Tables

**Table 1:** Description of selected malarial antigens.

	Sl. No.	Antigen ID	Common Name	ORF Fragment, (AAs)	MW, (kDa)
*P. falciparum* antigens	1	-	Green fluorescent protein (GFP)	1–238 (238)	26.9
	2	PF3D7_0930300	Merozoite surface protein1 (MSP1-19)	1612–1720 (108)	12.1
	3	PF3D7_0930300	Merozoite surface protein1 (MSP1-42)	1361–1720 (360)	41.5
	4	PF3D7_0930300	MSP1-42 V1	1361–1720 (360)	41.4
	5	PF3D7_0930300	MSP1-42 V2	1361–1720 (360)	41.6
	6	PF3D7_0930300	MSP1-42 V3	1361–1720 (360)	41.4
	7	PF3D7_0930300	MSP1-42 V4	1361–1720 (360)	41.4
	8	PF3D7_0930300	MSP1-42 V5	1361–1720 (360)	41.4
	9	PF3D7_0930300	MSP1-42 V6	1361–1720 (360)	41.3
	10	PF3D7_1133400	Apical membrane antigen 1 (AMA1)	22–544 (523)	60.5
	11	PF3D7_0424100	Reticulocyte binding protein homologue 5	1–526 (526)	63.0
South Asia *P. falciparum* top reactive serological markers [6]	12	PF3D7_0422100	Transmembrane emp24 domain-containing protein	1–385 (385)	45.0
	13	PF3D7_1002100	EMP1-trafficking protein	1–631 (631)	69.7
	14	PF3D7_0315400	Conserved protein, unknown function	1–256 (256)	30.5
	15	PF3D7_0620400	Merozoite surface protein10	1–525 (525)	61.2
	16	PF3D7_0935600	Gametocytogenesis-implicated protein	1–512 (512)	58.2
*P. vivax* antigens	17	PVX_099980	MSP1-19	1622–1729 (108)	11.8
	18	PVX_099980	MSP1-42	1325–1729 (428)	42.0
	19	PVX_092275	Apical membrane antigen 1 (AMA1)	43–487 (444)	50.1
South Asia *P. vivax* top reactive serological markers [6]	20	PVX_081830	Exported protein, unknown function	1–494 (496)	56.7
	21	PVX_116780	Protein transport protein SFT2, putative	1–268 (269)	29.0
	22	PVX_083560	Exported protein, unknown function	1–310 (311)	34.0
	23	PVX_121930	Exported protein, unknown function	1–289 (290)	32.4
	24	PVX_118705	Hypothetical protein, conserved	1–438 (439)	50.7

## Data Availability

The data presented in this study are available on request from the corresponding authors.

## Supplementary Materials

The following supporting information can be downloaded at: https://www.mdpi.com/article/10.3390/vaccines12010001/s1, Figure S1: *Plasmodium falciparum* MSP1-42 expression and characterization; Figure S2: Validation of bead fluorescence after defined protein attachment; Figure S3: Stability of antigens-adsorbed beads and their drifting; Table S1: Clinical summary of patient samples.
