# Supplementary material for: Multiplex Assays for Analysis of Antibody Responses to South Asian Plasmodium falciparum and Plasmodium vivax Malaria Infections
Source: Vaccines (Basel). 2023 Dec 19;12(1):1. doi: 10.3390/vaccines12010001 (PMC10818873; doi:10.3390/vaccines12010001)
Supplement: Supplementary file 1 [file vaccines-12-00001-s001.zip › vaccines-2666334-supplementary.pdf]

Supplemental Methods and Data:

Supplemental methods

Purification of Ctagged *Pf*MSP1-42 and GFP from wheat-translated lysates. The wheat cell-free system expressed antigens were purified using prepacked one ml CaptureSelect Ctag Affinity columns (ThermoFisher). The column was equilibrated with 20 mM Tris pH 7.5 100 mM NaCl, then passed through the wheat cell-free system translated lysate at 0.5 ml per min. The column was washed with 20 column volumes of 20 mM Tris pH 7.5, 100 mM NaCl, 0.05% Triton X-100, and 2 mM DTT. Bound antigen was eluted with 10 ml of elution buffer (20 mM Tris, 100 mM NaCl, 4 mM SEPEA peptide, pH 7.4). The eluted sample was buffer exchanged five times with Phosphate Buffer saline containing 0.05% TritonX-100, 2 mM DTT, and concentrated to a final volume of 0.2 ml using a 10K Amicon Ultra centrifugal filter (EMD Millipore, USA). Protein concentration was determined using a Bradford assay. The concentrated antigens were resolved on SDS-PAGE and visualized by Coomassie staining.

Antibody Production. Rabbit immunizations were contracted to GenScript (GenScript Biotech Corporation, NJ, USA). Rabbits (n=2) were immunized intramuscularly with 200 µg of C-tag purified *Pf*MSP1-42 protein formulated in Freund's complete adjuvant on day 0. Three booster immunizations of 200 µg, 400 µg, and 200 µg were carried out on days 14, 28 and 72. Booster immunizations were mixed with Freund's incomplete adjuvant. Total IgG was purified from rabbit sera using protein A column. Protein A purified antibodies were received from GenScript.

Preparation of *P. falciparum* 3D7 lysate. The P. falciparum 3D7 line was maintained in continuous culture using fresh erythrocytes at 2% hematocrit. A parasitemia of 5% was reached, and cultures were synchronized by two incubations in 5% sorbitol three hours apart. Cultures were maintained to the schizont stage, centrifuged down, and pellets were frozen at -80 °C. For parasite lysate preparation, pellets were thawed and washed twice with PBS containing a protease inhibitor cocktail (Roche, IN, USA). Saponin was added to a final concentration of 0.15% and incubated for two minutes at room temperature with gentle periodic tapping. The sample was centrifuged at 5000 x g for ten minutes, followed by three washes with PBS. A black pellet consisting of intact parasites minus RBC membrane was retained. To remove the parasite membrane, lysis buffer (0.05 M Tris pH 8.0, 5 mM EDTA, 0.5% TritonX-100, 0.2X Roche Protease Inhibitor) was added to the pellet and incubated on ice for two hours. The tube was vortexed every 15 minutes. The Total, soluble, and pellet fractions of the lysed sample were generated by centrifuging at 20,000 x g for 10 minutes. The samples were aliquoted and stored at -20°C.

Western Blot. Total, soluble, and pellet fractions of parasite lysate were resolved on a 12-4% gradient SDS-PAGE gel. The resolved proteins from the gel were electrophoretically transferred to the Immobilon PVDF membrane at 35V for two hours at 4 °C in a transfer buffer (0.025 M Tris base, 0.2 M glycine, 0.002M SDS). After blocking the PVDF membrane with 5% milk in wash buffer (wash buffer: 0.15 M NaCl, 0.02 M Tris HCl, 0.005 M Tris base, 0.9 mM Tween20), the membrane was blotted with rabbit anti-PfMSP1-42 antibody (1:20,000). LiCor IRDye800CW conjugated goat anti-rabbit (1:5000) was used as the secondary antibody. The PVDF membrane was directly scanned. Blots were scanned at 680 and 780nm on a LiCor Odyssey gel imaging scanner.

Growth Inhibition Assay. The *P. falciparum* 3D7 line was maintained in continuous culture using fresh erythrocytes at 0.5% hematocrit and synchronized by two incubations in 5% sorbitol three hours apart. Synchronized early/mid trophozoites were adjusted to 0.5% parasitemia and then incubated in triplicates with Phosphate Buffer Saline (PBS) containing various concentrations of rabbit anti-*Pf*MSP1-42IgG or PBS alone in 96 well plates (tested in triplicate). Final parasitemia was determined by SYBR Green I staining. Briefly, an equal volume of parasite culture was incubated with an equal volume of 2X SYBR Green I stain (in PBS) for 20 minutes in the dark. After incubation, the total volume in wells was brought to 200 µl. A replicate plate was made with a 1/5 dilution of the original plate. To avoid clogging and obtaining inaccurate single-cell counts on the flow cytometer, an optimized protocol from the lab involving 1/5 dilution of the original plate was performed. Thirty thousand events per sample were read by flow cytometry (Accuri BD C6, BD Biosciences, Franklin Lakes, NJ). Events were converted to percent parasitemia, and then percent proliferation was determined by dividing the percent parasitemia of trend/percent parasitemia from PBS alone control.

Stability and drift of adsorbed antigens on Anti-Ctag functionalized beads (AFBs). A stability assay was conducted to assess the longevity of antigens adsorbed onto the anti-Ctag conjugated beads and the chance of drifting the antigens between beads. In these studies, three sets of AFBs adsorbed with *Pf*MSP1-42, GFP, or no antigen were mixed and multiplexed against anti-*Pf*MSP1 rabbit antibodies as well as P8 sera (**Figure S3**). The observed fluorescence was recorded as day 1 MFIs. Mixed beads were stored in the dark at 4°C for 90 days. The multiplexing experiment was repeated on day 90 to record any change in MFI.

Supplemental data

Figure S1

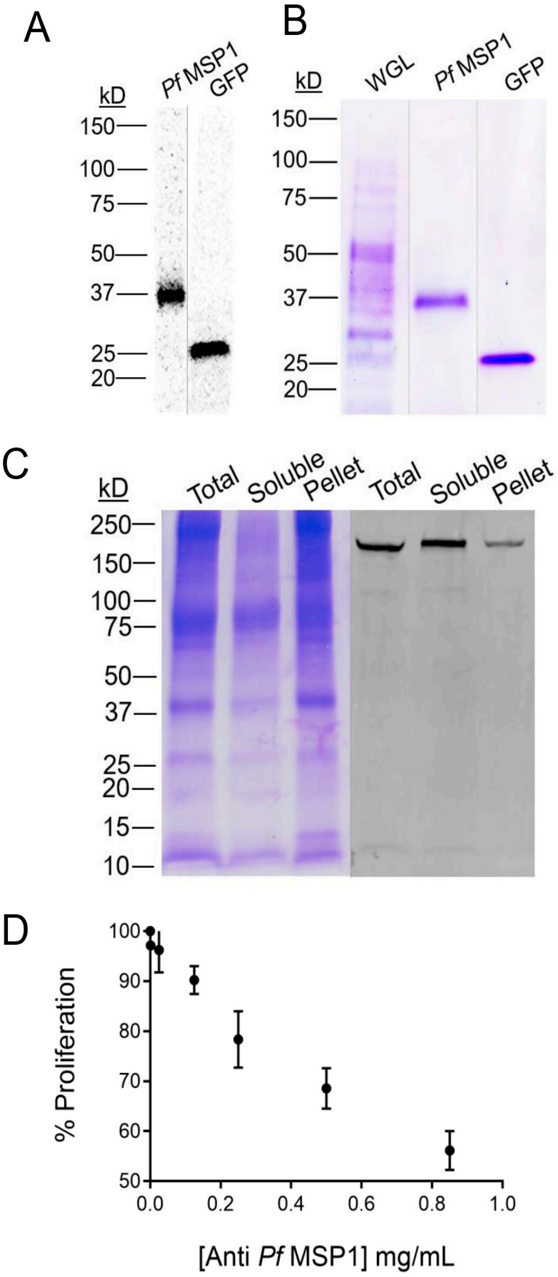

S1. *Plasmodium falciparum* MSP1-42 expression and characterization. (A) Autoradiogram of 14C-Leu labeled Ctagged *Pf*MSP1 and GFP expressed in wheat germ cell-free system. (B) Demonstration of purity of affinity column purified Ctagged *Pf*MSP1 and GFP on a Coomassie-

stained gel. (C) Validation of anti-*Pf*MSP1 antibody specificity against *P. falciparum* parasite lysates. (D) Growth inhibition assay of *P. falciparum* with anti-*Pf*MSP1 antibodies.

Figure S2

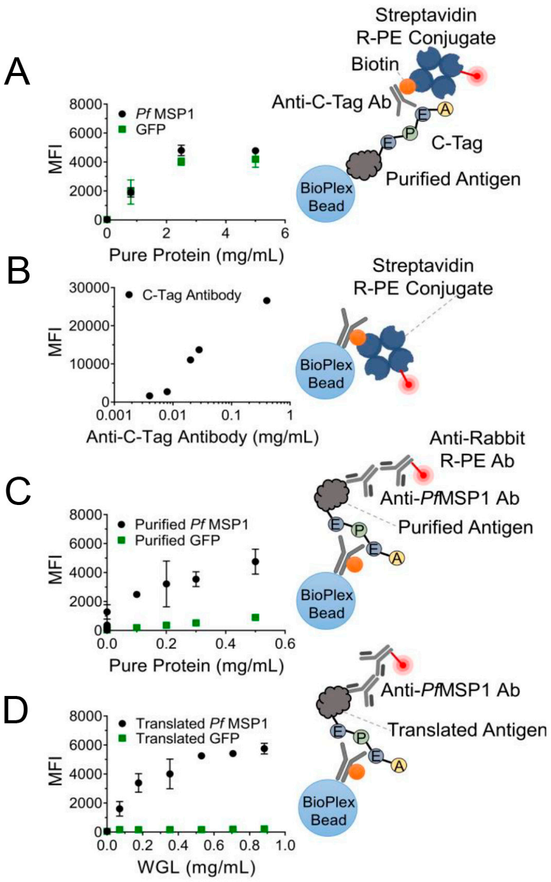

Figure S2. Validation of bead fluorescence after defined protein attachment. (A) Titration of chemical attachment of purified Ctagged *Pf*MSP1 and GFP onto BioPlex beads. (B) Saturated chemical attachment of biotinylated anti-Ctag antibodies to beads. (C and D) The antigen adsorption capacity of anti-Ctag antibody-coated beads. Anti-Ctag antibody-coated beads were tested for their binding capacity from purified (C) and from translated lysates (D) of *Pf*MSP1 and GFP

Figure S3

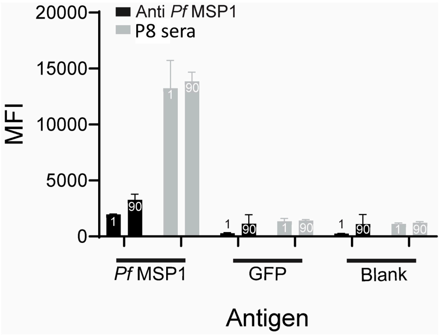

Figure S3. Stability of antigens-adsorbed beads and their drifting. No change in MFI for *Pf*MSP1-42 over 90 days when probed with rabbit antibody or P8 antibody indicates that the antigen is intact and stable on the bead. Similarly, no decrease of MFI for the *Pf*MSP1-42 adsorbed beads (*Pf*MSP1), no increase of MFI for GFP-adsorbed beads (GFP), or beads with no antigen (blank) over 90 days when probed with rabbit antibody or P8 antibody indicates that there is no exchange of adsorbed antigens between the beads.

Supplementary Table S1. Clinical summary of patient samples

Demographics				MESA Lab Information								Hospital Information																					Clinical Classification					
P ID	Gender	Age	Temperature	RDT	Thick Smear	PCR	% Parasitemia	Parasite Density	HRP2	% Gametocytemia	Gametocyte Density	Inpatient	Days in Hospital	Glasgow coma - Eye (Lowest)	Glasgow coma - Verbal (Lowest)	Glasgow coma - Motor (Lowest)	Glasglow coma - Total (Lowest)	Altered Consciousness	Hb (Lowest)	HCT (lowest)	Blood Glucose (lowest)	Blood urea nitrogen (highest)	Serum Creatinine (highest)	Abnormal bleeding	Respiration Rate (highest)	PO2 (lowest)	Total bilirubin (highest)	Pulmonary oedema	Plasma Biocarbonate (lowest)	Systolic BP (lowest)	Platelets (lowest)	Death	Cerebral Malaria	Severe Malaria	Degree of Severity	Uncomplicated, Hospital Malaria, High Fever	Uncomplicated, Hospital Malaria	Outpatient Malaria
P1	M	15	103.8	Pf	Pf	Pf	2.4	3542	4.45	0	437	N																										X
P2	M	17	107	Pf	Pf	Pf	0.65	4360	11.64	0.25	880	N																										X
P3	M	22	99.4	Pf	Pf	Pf	1	3067	119.63	0	310	Y	4	4	5	5	14	N	9.5	28	M	49	0.9	N	18	M	2.4	N	M	80	120000	M		X	1			
P4	M	26	105.5	Pf	Pf	Mixed	1.91	5372	29.54	0	1353	N																										X
P5	M	27	97.8	Pf	Pf	Pf	3.6	7842	347.85	0	0	Y	1	2	3	3	8	M	7.5	22	M	212	5.7	Y	M	54.4	11.7	N	8.1	149	30000	Y	X	X	6			
P6	M	35	98.8	Pf	Pf	Pf	0.1	660	nd	0	0	Y	8	4	5	5	14	N	12.7	40	89	36	1.2	N	28	M	2	N	M	100	220000	N		X	1			
P7	F	37	100	Pf	Pf	Pf	1.3	8238	15.86	0	515	Y	8	4	5	5	14	M	6.6	21	95	32	0.7	M	16	M	7	M	M	90	40000	N		X	1			
P8	M	50	99.7	Pf	Pf	Pf	0.2	1307	21.32	0	155	N																										X
P9	M	60	105	Pf	Pf	Pf	2	8078	62.9/104.27	0	157	Y	7	4	5	5	14	N	9.2	20.1	95	92	1.7	N	20	M	19.5	N	M	110	70000	N		X	2			
P10	M	60	106	Pf	Pf	Pf	1.9	3200	44.08/306.31	0.25	280	Y	10	2	2	4	8	N	5.5	15.6	21	72	1.4	N	36	72.3	10.9	N	15.3	110	310000	Y	X	X	5			

Mixed, both Pf and Pv infected; Y, yes; N, no; M, missing data
